# Long-Term Arthralgia after Mayaro Virus Infection Correlates with Sustained Pro-inflammatory Cytokine Response

**DOI:** 10.1371/journal.pntd.0004104

**Published:** 2015-10-23

**Authors:** Felix W. Santiago, Eric S. Halsey, Crystyan Siles, Stalin Vilcarromero, Carolina Guevara, Jesus A. Silvas, Cesar Ramal, Julia S. Ampuero, Patricia V. Aguilar

**Affiliations:** 1 Institute for Human Infections and Immunity, Galveston, Texas, United States of America; 2 Department of Pathology, University of Texas Medical Branch, Galveston, Texas, United States of America; 3 US Naval Medical Research Unit No. 6, Lima, Peru; 4 US Naval Medical Research Unit No. 6, Iquitos, Peru; 5 Hospital Regional, Loreto, Peru; 6 Center for Biodefense and Emerging Infectious Diseases, Galveston, Texas, United States of America; Florida Department of Health, UNITED STATES

## Abstract

Mayaro virus (MAYV), an alphavirus similar to chikungunya virus (CHIKV), causes an acute debilitating disease which results in the development of long-term arthralgia in more than 50% of infected individuals. Currently, the immune response and its role in the development of MAYV-induced persistent arthralgia remain unknown. In this study, we evaluated the immune response of individuals with confirmed MAYV infection in a one-year longitudinal study carried out in Loreto, Peru. We report that MAYV infection elicits robust immune responses that result in the development of a strong neutralizing antibody response and the secretion of pro-inflammatory immune mediators. The composition of these inflammatory mediators, in some cases, differed to those previously observed for CHIKV. Key mediators such as IL-13, IL-7 and VEGF were strongly induced following MAYV infection and were significantly increased in subjects that eventually developed persistent arthralgia. Although a strong neutralizing antibody response was observed in all subjects, it was not sufficient to prevent the long-term outcomes of MAYV infection. This study provides initial immunologic insight that may eventually contribute to prognostic tools and therapeutic treatments against this emerging pathogen.

## Author Summary

Mayaro virus (MAYV) causes an acute debilitating disease which results in the development of long-term arthralgia in more than 50% of infected individuals, similarly to what has been described with CHIKV. In this study, we evaluated the immune response of individuals with confirmed MAYV infection in a one-year longitudinal study carried out in Loreto, Peru. Here, we report that MAYV infection elicits robust immune responses that result in the development of a strong neutralizing antibody response and the secretion of pro-inflammatory immune mediators. These inflammatory mediators, in some cases, differed to those observed by others for CHIKV. We also observed a strong neutralizing antibody response in all the study subjects; however, this response was not sufficient to prevent the long-term outcomes of MAYV infection. Taken together, this study provides initial immunologic insight that may eventually contribute to the development of prognostic tools and potential therapeutic treatments against this emerging pathogen.

## Introduction

Alphaviruses are pathogens responsible for endemic human diseases in various regions throughout the world [1, 2]. They can be transmitted to humans by mosquitoes of the genera *Ochlerotatus*, *Aedes*, *Psorophora*, *Mansonia*, *Haemagogus*, and *Culex*, among others [3, 4]. Alphavirus infection can result in severe disease, such as encephalitis (primarily caused by New World alphaviruses), or incapacitating chronic disease, such as persistent arthralgia and myalgia (primarily caused by Old World alphaviruses) [5, 6]. The global distribution of some of the alphavirus vector species, the low population immunity, and the severe disease induced by infection underscore the potential of these viruses to emerge as significant human pathogens.

Mayaro virus (MAYV), the etiological agent of Mayaro fever (MAYF), belongs to the group of arthritogenic alphaviruses that also includes CHIKV, Ross River (RRV), Sindbis, Barmah Forest, and O’nyong-nyong viruses [7–10]. MAYV was first isolated in 1954 in Trinidad and, since then, it has been isolated in a number of countries in tropical South America, including Trinidad, Bolivia, French Guiana, Peru, Venezuela and Brazil [10–15].

MAYV is presumably transmitted primarily by tree-dwelling *Haemagogus* mosquitoes [4]. However, laboratory evidence suggests that *Ae*. *aegypti* mosquitoes can serve as competent vectors [16]. Interestingly, this scenario parallels that which led to the emergence of CHIKV in the Eastern Hemisphere and to the expansion and emergence of CHIKV in other regions, including the Western Hemisphere in 2014 [17]. Thus, the potential for MAYV to emerge as a global pathogen cannot be overlooked.

MAYF is clinically characterized by a number of non-specific symptoms such as high fever, rash, myalgia, headache, and arthralgia. We have previously reported that 54% of MAYV infected patients develop persistent arthralgia affecting the major joints [18]. Despite this knowledge, it is currently unknown if MAYV infection induces a pro-inflammatory cytokine response similar to that described for other arthritogenic alphaviruses [19–23]. Furthermore, the role of the immune response in MAYV induced persistent arthralgia remains uncertain.

In this study, we evaluated the immune response of individuals with MAYF in a one-year longitudinal study carried out in Peru. Specifically, our objective was to assess whether the magnitude of the neutralizing antibody responses and cytokine profiles differed among: 1) subjects with MAYF who went on to suffer persistent arthralgia, 2) subjects with MAYF who did not develop persistent arthralgia, and 3) healthy controls.

## Materials and Methods

### Ethical Statement

This study was approved by the Ministry of Health of Peru and by the Institutional Review Boards (IRBs) of the U.S. Naval Medical Research Unit No. 6 (NMRCD.2010.0010) and University of Texas Medical Branch (12–133). Written informed consent forms were obtained from all participants prior to study enrollment and sample collection.

### Study Cohort and Sample Collection

The study cohort and sample collection have been previously described for 16 subjects [18]. Five additional subjects have been included in this study. Briefly, subjects over five years of age with febrile illness (oral temperature ≥38°C) were recruited from health centers in Iquitos, a city of about 380,000 inhabitants located 120 meters above sea level, and Yurimaguas, a city of about 70,575 inhabitants located 106 meters above sea level in the Amazon Basin in northeastern Peru. Blood samples were collected at the time of enrollment (acute) and at follow up visits 20 (±10), 90 (±10), 180 (±15), and 360 (±30) days post enrollment. MAYV infection was confirmed by viral isolation, RT-PCR against a 784 nucleotide stretch within the E2 and E3 portion of the genome, or IgM seroconversion. Seroconversion was defined as a ≥4-fold increase in IgM titer between the acute and the convalescent (day 20) visit. Day of illness at enrollment and clinical outcome during the first 12 months after MAYV infection are depicted in Table 1.

**Table 1:** Neutralizing antibody titers to alphaviruses and disease profile of study patients.

Subject	Enrollment date	Days of illness at enrollment	MAYV	VEEV	EEEV	Persistent arthralgia*
1	1/25/2013	1	160	<20	<20	Yes
2	3/19/2013	2	640	<20	<20	Yes
3	2/21/2013	1	320	<20	<20	No
4	4/23/2012	4	640	<20	<20	Yes
5	1/17/2012	3	320	<20	<20	Yes
6	1/31/2011	2	320	<20	<20	Yes
7	4/11/2011	2	160	<20	<20	No
8	4/13/2011	2	1280	40	<20	No
9	4/13/2011	2	320	<20	<20	No
10	7/5/2011	1	1280	<20	<20	No
11	8/10/2011	1	160	<20	<20	Yes
12	8/11/2011	3	640	<20	<20	Yes
13	4/13/2011	4	160	<20	<20	No
14	4/20/2011	2	640	20	<20	No
15	8/11/2011	2	160	<20	<20	Yes
16	1/15/2011	1	640	<20	<20	Yes
17	2/28/2011	2	160	<20	<20	Yes
18	5/25/2011	3	320	<20	<20	Yes

Plaque reduction neutralization tests were carried-out in convalescent phase samples collected at follow up visit 20 (±10) days after Mayaro (MAYV) infection. EEEV = Eastern equine encephalitis virus; VEEV = Venezuelan equine encephalitis virus.

*Persistent arthralgia was defined as the presence of arthralgia for at least three months after the acute presentation.

### Healthy Donor Samples

Blood samples were collected from seven healthy subjects residing in Iquitos, Peru. All samples were seronegative to alphaviruses as determined by a previously described ELISA IgG [24]. S1 Fig details the cytokine and chemokine values obtained with the healthy subject group from the Amazon basin of Peru.

### Bio-Plex Human Cytokine Assay

Samples were assayed for the presence of 23 cytokines and chemokines, including IL-1Ra, IL-2, IL-4, IL-5, IL-6, IL-7, IL-8, IL-9, IL-10, IL-12p70, IL-13, IL-17, basic fibroblast growth factor (FGF), eotaxin, granulocyte colony stimulating factor (G-CSF), granulocyte macrophage colony stimulating factor (GM-CSF), interferon gamma (IFN-γ), monocyte chemoattractant protein-1 (MCP-1), macrophage inflammatory protein (MIP)-1α, MIP-1β, platelet-derived growth factor (PDGF)-BB, tumor necrosis factor alpha (TNF-α), and vascular endothelial growth factor (VEGF) according to the manufacturer’s recommendations (BioRad, Hercules, CA). Results were analyzed through a BioPlex 2000 instrument equipped with BioManager analysis software (BioRad). Samples with cytokine concentrations below the level of detection were arbitrarily assigned a set value one decimal unit below the lowest standard range for the corresponding cytokine.

### Viruses

Working stocks of each MAYV, VEEV subtype ID, and EEEV were generated in Vero 76 cells. Viral handling and experimentation requiring the use of VEEV and EEEV were performed in a BSL-3 facility following BSL-3 safety precautions. The virus strains were initially isolated from febrile patients residing in Peru or from mosquito pools collected in Loreto, Peru [1, 25]. Working viral stocks were titered by plaque assay, as previously described [26].

### Plaque Reduction Neutralization Tests (PRNTs)

PRNTs were performed as previously described [26]. Briefly, samples were diluted 1:10 followed by incubation at 56°C for one hour. Two-fold serial dilutions of the inactivated samples were incubated with approximately 50 plaque forming units of virus. The sample/virus solution was mixed thoroughly, the plates were then sealed and incubated overnight at 4°C, and then added to the corresponding well of a 12-well plate containing a confluent monolayer of Vero 76 cells. Virus adsorption was allowed to take place at 37°C for one hour. Next, an agar overlay was added to the wells. The plates were then incubated at 37°C for 36–48 hours to allow for plaque formation and stained for 30 minutes with 0.5% crystal violet in methanol. Following the incubation period, the plates were washed and the plaques were counted. Neutralization titers were considered as the highest serum dilution that reduced plaque formation by ≥ 80%.

### Statistical Analysis

Data sets pertaining to the different phases of MAYF as well as the different patient groups were analyzed by 2-tailed Mann-Whitney test. The group comparisons included: (a) subjects with MAYF versus healthy donors, (b) subjects with MAYF and no persistent arthralgia versus healthy donors, (c) subjects with MAYF and persistent arthralgia versus healthy donors, and (d) subjects with MAYF and no persistent arthralgia versus subjects with MAYF and persistent arthralgia. For groups (c) and (d), persistent arthralgia was defined as presence of arthralgia for at least three months after the acute presentation. In addition, data sets were evaluated by using Wilcoxon non-parametric tests to evaluate the evolution of cytokine levels at each time point post-infection. Data were considered significant when p-values were lower than 0.05. All statistical analysis was performed using GraphPad Prism version 6 software (La Jolla, CA).

## Results

### The Magnitude of the Anti-MAYV Neutralizing Antibody Response Is Not Associated with the Development of Chronic Arthralgia following MAYV Infection

We previously reported that 54% of subjects in our cohort developed chronic joint pain that lasted for at least one year following MAYV infection [18]. To determine if the magnitude of the anti-MAYV neutralizing antibody response correlates with protection against long-term arthralgia, we performed plaque reduction neutralization testing on samples collected during the convalescent phase (day 20±10 days) of the disease (Table 1). In our cohort, 100% of the MAYV infected subjects developed MAYV neutralizing antibodies. The anti-MAYV neutralizing antibody titers ranged from 160–1280 with a median value of 320. However, no association between the magnitude of the neutralizing antibody titers and the development of chronic arthralgia were observed (mean antibody titers ±SD in subjects with persistent arthralgia and those with no persistent arthralgia were 378±217 vs. 594±495, respectively; p>0.05).

### Exposure to Other Endemic Alphaviruses in the MAYF Study Cohort

Antibody mediated cross-protection by related alphaviruses has been reported in animal models of alphavirus infection [27–30]. In Peru, there are two other alphaviruses that are known to cause human disease (albeit they belong to a serological complex distinct from MAYV): VEEV and EEEV. Epidemiological investigations have found a VEEV and EEEV seroprevalence rate of 25% and 3%, respectively in the Amazon basin of Peru [31, 32]. In order to determine if our cohort had prior exposure to these alphaviruses, and thus potentially harbor a cross-reactive protective immune component, we performed PRNTs against VEEV and EEEV on the day 20 samples. It should be noted that PRNTs was chosen to evaluate exposure to alphaviruses because the assay is highly specific and therefore it allows a clear determination of exposure to a particular alphavirus without the concern of false positive results. By PRNT, we observed that two subjects (11%) had anti-VEEV neutralizing antibodies; neither subject developed chronic arthralgia (Table 1). No neutralizing antibodies against EEEV were detected in our cohort.

### Profile of Immune Mediators following MAYV Infection

**Fig 1:**
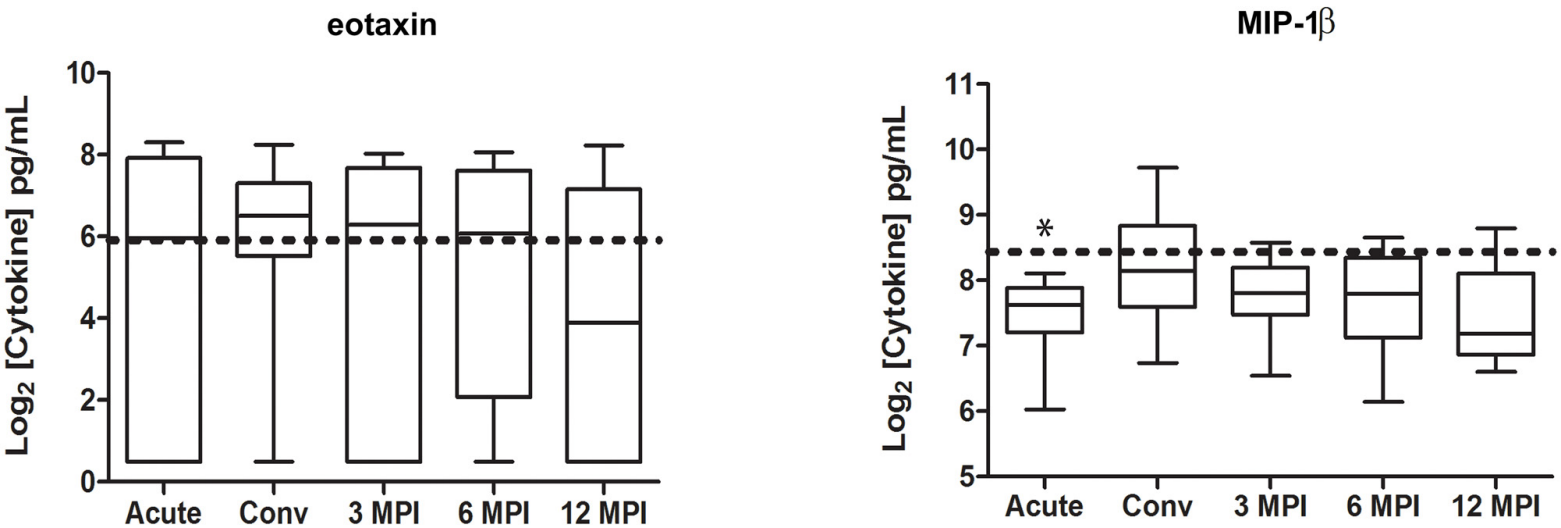
Kinetic profile of cytokines and chemokines similar to the concentrations detected in healthy donor controls following MAYV infection. Serum samples were collected at the acute visit as well as the convalescent visit (20±10 days), at 90±10 days, at 180±15 days, and at 360±30 days after the acute visit. The serum concentrations of a variety of cytokines were assessed by a multiplex cytokine bead array. The box plot denotes the median, 25^th^ percentile, and 75^th^ percentile cytokine levels. The whiskers denote the minimum and maximum cytokine levels observed at each time point. The horizontal dotted line represents the median cytokine values for the healthy donor controls. Comparisons between MAYV infected subjects and healthy donors were performed by a 2-tailed Mann-Whitney test (*p<0.05).

**Fig 2:**
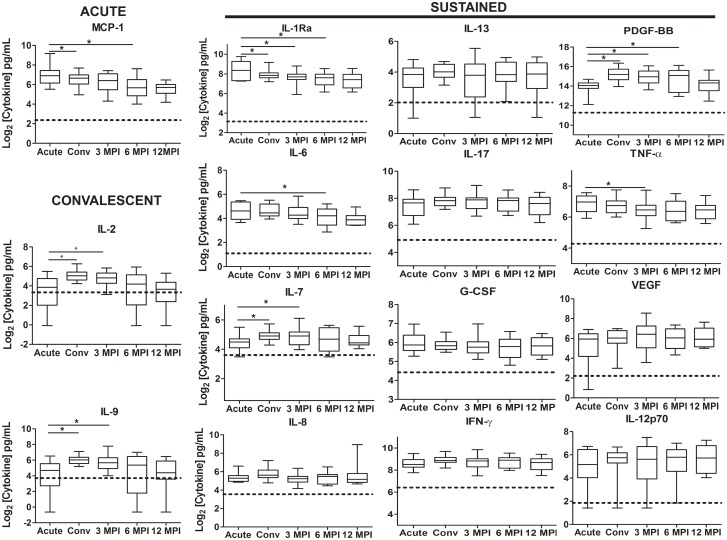
Kinetic profile of cytokines and chemokines significantly affected following MAYV infection. Serum samples were collected at the acute visit as well as the convalescent visit (20±10 days), at 90±10 days, at 180±15 days, and at 360±30 days after the acute visit. The serum concentrations of a variety of cytokines were assessed by a multiplex cytokine bead array. Three kinetic profiles are presented based on the peak of cytokine expression: **Acute:** cytokines/chemokines that peak during the acute phase (upon enrollment); **Convalescent:** cytokines/chemokines that peak during the convalescent phase 20 (±10) days post enrollment and may remain elevated for few months after MAYV infection; and **Sustained:** cytokines/chemokines whose expression remain elevated up to 12 months following MAYV infection. The box plot denotes the median, 25^th^ percentile, and 75^th^ percentile cytokine levels. The whiskers denote the minimum and maximum cytokine levels observed at each time point. The horizontal dotted line represents the median cytokine values for the healthy donor controls. Data sets were evaluated by using Wilcoxon non-parametric tests to evaluate the evolution of cytokine levels at each time point post-infection (*p<0.05).

Next, we sought to investigate the profile of several immune mediators following MAYV infection and observed that: 1) Some cytokine and chemokine levels were similar to the concentrations detected in healthy donor controls (Fig 1), or significantly below those observed in healthy donor controls (p<0.05)(S2 Fig). 2) The chemokine MCP-1, which regulates migration and infiltration of monocytes/macrophages, peaked during the acute phase of MAYV infection and was significantly elevated when compared to the levels observed in the healthy donor control group as well as those observed during the convalescent phase, and 6 months post-infection (p<0.05) (Fig 2). 3) The cytokine levels of IL-2 and IL-9, which are involved in the stimulation of cell proliferation, were significantly elevated during the convalescent phase, and remained elevated 3 months post-infection (Fig 2). 4) A group of cytokines and chemokines that were significantly elevated when compared to the healthy donor controls (p<0.05) at most of the analyzed time points. Immune mediators in this group include: IL-1Ra, IL-6, IL-7, IL-8, IL-13, IL-17, G-CSF, IFN-γ, PDGF-BB, TNF-α, VEGF, and IL-12p70 (Fig 2).

### Comparison of Immune Mediators among Subjects Who Developed Persistent Arthralgia, Those That Fully Recovered, and a Healthy Control Group

**Fig 3:**
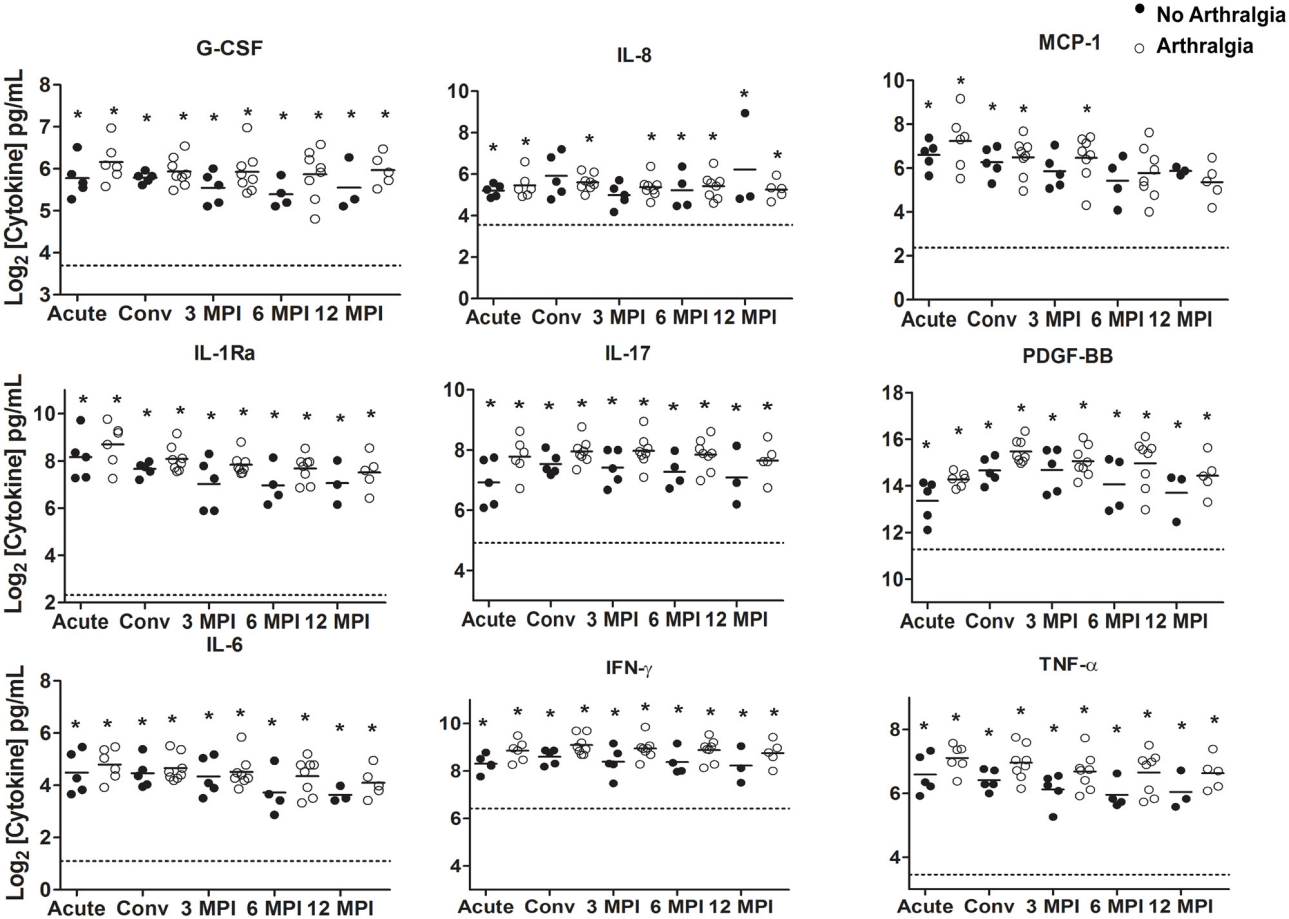
Induction of cytokine/chemokine immune mediators in MAYV-infected subjects with or without persistent arthralgia. Serum samples were collected at the acute visit as well as the convalescent visit (20±10 days), at 90±10 days, at 180±15 days, and at 360±30 days after the acute visit. The serum concentrations of a variety of cytokines were assessed by a cytokine bead array. The cytokine concentrations in subjects with (open circles) or without (closed circles) persistent arthralgia were compared to healthy donor controls by a 2-tailed Mann-Whitney test. An asterisk (*) indicates statistical significance (p<0.05) when compared to healthy controls. Comparisons between MAYV infected groups were also performed by a 2-tailed Mann-Whitney test. No statistical significance (p<0.05) between the MAYV infected groups was found. The horizontal dotted line represents the median cytokine values for the healthy donor controls. Each circle corresponds to the cytokine concentration of an individual subject and the solid horizontal line represents the mean cytokine concentration of the group.

**Fig 4:**
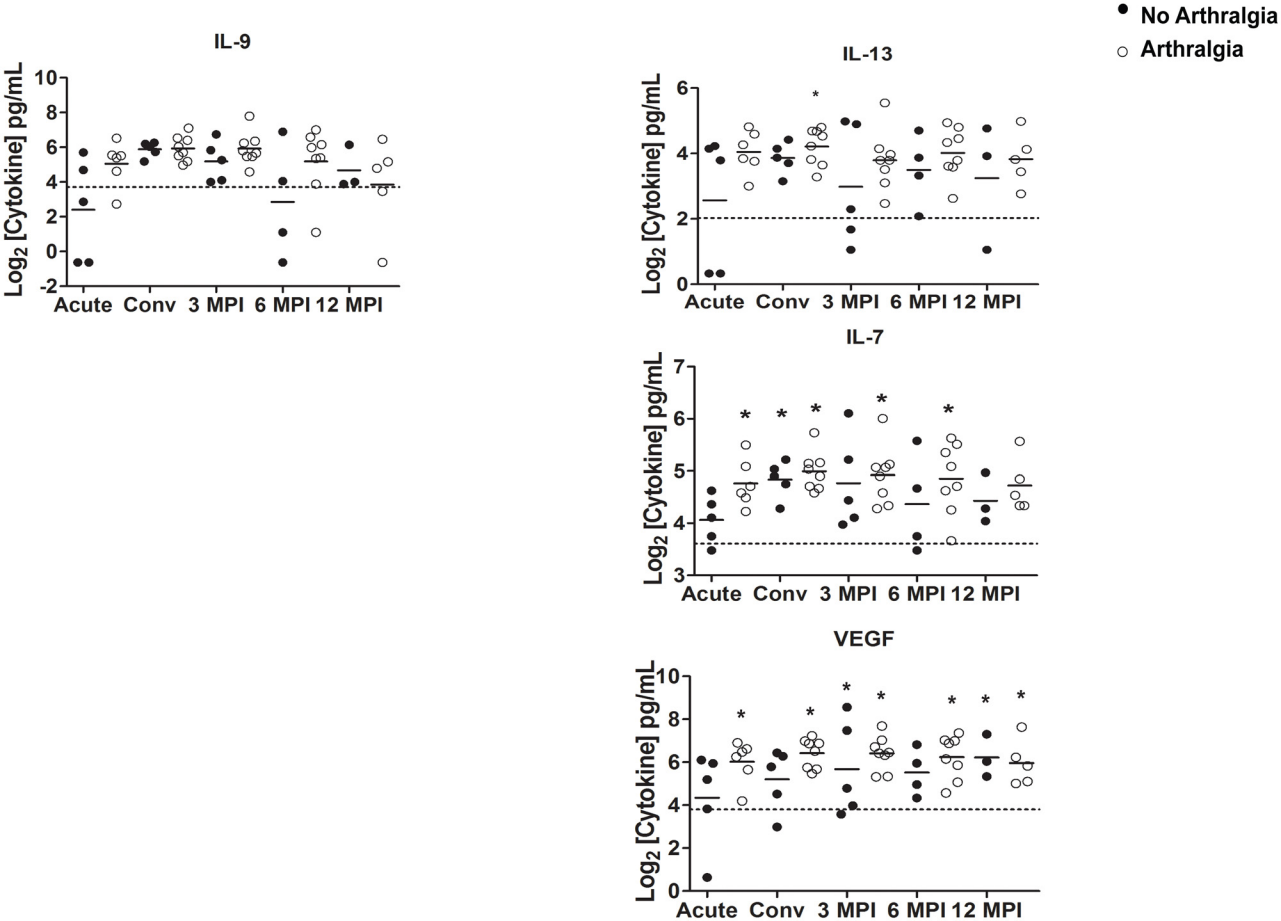
Cytokine/chemokine immune mediators in MAYV-infected subjects and potential predictors of MAYV-induced long-term arthralgia. At the acute visit as well as the convalescent visit (20±10 days), at 90±10 days, at 180±15 days, and at 360±30 days after the acute visit, cytokine concentrations of subjects with (open circles) or without (closed circles) persistent arthralgia were compared to healthy donor controls as well as between MAYV-infected groups by a 2-tailed Mann-Whitney test. An asterisk (*) indicates statistical significance (p<0.05) when compared to healthy controls. The horizontal dotted line represents the median cytokine values for the healthy donor controls. Each circle corresponds to the cytokine concentration of an individual subject and the solid horizontal line represents the mean cytokine concentration of the group. Levels of IL-9 were not significantly different when compared to the healthy control group. Immune mediators on the right are potential predictors of MAYV-induced long-term arthralgia.

The cytokine and chemokine levels of G-CSF, IL-1Ra, IL-8, IL-17, IFN-γ, MCP-1, PDGF-BB and TNF-α remained significantly and consistently elevated in subjects who developed persistent arthralgia and those that fully recovered when compared with the healthy control group at all, or most of the analyzed time points (p<0.05) (Fig 3). In contrast, the cytokine levels of IL-9 were not significantly different at the time points examined when compared to healthy donors (p>0.05) (Fig 4). We also observed that the immune mediator IL-13 was significantly elevated in subjects developing persistent arthralgia during the convalescent phase of the disease, when compared to healthy donors. In contrast, in subjects that fully recovered from MAYV infection this mediator was not significantly different to healthy donor controls (Fig 4). Similarly, we observed that in subjects developing persistent arthralgia, the levels of IL-7 and VEGF were significantly elevated at most or all of the time points examined when compared to healthy donors whereas in subjects that fully recovered from MAYV infection, they were only significantly induced during the convalescent phase (IL-7) or 3 and 6 months post-infection (VEGF)(Fig 4). Overall, we observed a tendency for higher cytokine concentrations of the majority of the cytokines measured in the subjects developing persistent arthralgia versus subjects fully recovering after MAYV infection; however, these differences were not statistically significant.

## Discussion

MAYV continues to be an important cause of febrile illness in South America [1, 33]. MAYV infections are usually associated with a highly debilitating disease characterized by fever, headache, diarrhea, vomiting, myalgia, arthralgia and rash [18]. Similar to chikungunya fever (CHIKF), severe joint pain can persist for months or years following MAYF [14, 18]. Alphavirus infections typically result in the production of long lasting neutralizing antibodies that remain detectable several years after infection [34], and past studies have demonstrated the role for both neutralizing and non-neutralizing antibody mediating protection and/or recovery from alphavirus infection [35]. In this study, we found that all the subjects in our cohort were able to develop robust anti-MAYV neutralizing responses (median of 320); however, the magnitude of this response did not differ between subjects that developed persistent arthralgia and those that fully recovered, suggesting that neutralizing antibodies alone are unable to fully protect individuals from the development of persistent arthralgia. This information should be taken into consideration as new vaccines against MAYV and other arthritogenic alphaviruses are designed and tested for efficacy. The fact that development of a robust neutralizing antibody response is not sufficient to protect against chronic disease might be due to the inability of the antibodies to reach and neutralize viruses that may locate in various joints. While the neutralizing antibody response did not protect against persistent arthralgia after MAYV infection, other studies have found that the isotype and timing of the neutralizing antibodies, in particular IgG3, is associated with protection against CHIKV-induced persistent arthralgia [36]. Thus, it remains to be determined if there is a link between the IgG isotype and the development of persistent arthralgia after MAYV infection. It was also previously shown that a subset of patients suffering from a rheumatoid arthritis (RA)-like illness following CHIKV infection possessed elevated levels of CHIKV-specific IgM that persisted for up to 6 months following infection [23, 37, 38]. However, in our cohort, while elevated at day 20 post-infection, anti-MAYV IgM antibodies were undetectable after 3 months [18]. It is also possible that alphavirus infection may lead to the generation of autoantibodies that can lead to joint damage. One study has reported the presence of anti-cyclic citrullinated peptide (ccp) antibodies, which has been used as a diagnostic and prognostic tool for rheumatoid arthritis, in patients suffering from CHIKF [39]. However, the presence of anti-ccp antibodies was detected in less than 30% of patients that had suffered from CHIKF and therefore it is still unclear if these antibodies contribute to CHIKV pathogenesis. Furthermore, it is currently unknown if these autoantibodies are developed following MAYV infection.

Alphavirus infection results in the induction of a robust inflammatory response in both humans and animals [19–23, 40]. The acute phase of CHIKV infection is characterized by elevated concentrations of pro-inflammatory innate immune factors such as IL-6, IL-7, IL-8, IL-12p70, IL-15, IP-10, and MCP-1, among others [20–23, 41]. During the chronic phase, the immune response is characterized by elevated concentrations of IL-1β, IL-5, IL-10, IL-12p70, IL-17, IFN-γ and TNF-α [20–23]. Longitudinal studies assessing the serological immune response following CHIKV infection have identified immune mediators, such as IL-1β, IL-6, and GM-CSF, as predictors of disease severity [20, 21, 42]. Our findings suggest that the immune response following MAYV infection, much like that induced by CHIKV, is predominantly inflammatory during the acute phase. However, the composition of elicited immune mediators differed between CHIKV and MAYV. Of particular interest are the low/baseline concentrations of GM-CSF, IL-5, and IL-10 following MAYV infection, which may point towards differences between CHIKV and MAYV in inducing the activation of specific immune cell populations.

In our cohort, we observed that MAYV-infected individuals have high levels of MCP-1 during the acute phase of the disease when compared to the healthy control group whereas IL-2 and IL-9 were predominantly elevated during the convalescent phase. We also observed significantly elevated levels of IL-13 in subjects with persistent arthralgia (but not in subjects that fully recovered) when compared to healthy subjects suggesting that IL-13 could potentially serve as predictor of severe outcome after MAYV infection. Furthermore, the levels of IL-7 and VEGF were also significantly elevated at all the time points examined in subjects with persistent arthralgia whereas they were elevated only during the convalescent phase (IL-7) or 3 and 12 months post-infection (VEGF) in subjects that fully recovered from the disease. These data suggest that these markers can be considered as predictors of severe outcome at early stages of the disease. These observations are also important because VEGF is known to have pro-inflammatory and anti-apoptotic roles in RA pathogenesis whereas IL-7 contributes to chronic inflammation and joint destruction [43–45]. Therefore, the contributions of these immune mediators to MAYV pathogenesis deserve to be further explored. It was also noted that the cytokine levels of IL-17 were elevated in subjects with or without persistent arthralgia at all the time points analyzed when compared to the healthy control group. IL-17 has been linked to joint inflammation, joint destruction, and cartilage/bone degradation in *in vitro* and *in vivo* studies [46]. In addition, IL-17 is elevated in patients suffering from RA, where the magnitude of the concentrations has been associated with disease severity [47]. IL-17 can induce the production of IL-6, which has been recently linked to bone loss following RRV infection [48]. Thus, the contribution of IL-17 to MAYV pathogenesis warrants further investigations.

In our study, elevated concentrations of PDGF-BB and IL-1Ra were observed for up to 12 months after MAYV infection. PDGF-BB is responsible for upregulating matrix metalloproteinases in joint synovia and promoting joint repair. In addition, PDGF-BB can suppress IL-1β induced cartilage degradation [49]. Moreover, the anti-inflammatory protein IL-1Ra has been shown to be upregulated in joints of patients with RA and was found to be effective in reducing joint destruction in experimental RA models [50]. Thus, it is possible that the sustained upregulation of PDGF-BB and IL-1Ra is due in part as an effort to combat the pathological effects of joint inflammation. It should also be noted that not all the anti-inflammatory cytokines were induced after MAYV infection. In particular, the cytokine levels of IL-10 were significantly below those detected in the healthy subject group.

Our study has provided the first longitudinal overview of the immune responses elicited following MAYV infection. Collectively, we observed that the serological response to MAYV infection and the subsequent development of persistent arthralgia is associated with the induction of a robust inflammatory response, similar to what has been observed with CHIKV infection. However, there are sufficient differences between the types of immune mediators driving the inflammatory responses to warrant a more careful examination of the mechanisms of MAYV-induced pathology. Only then could treatments be tailored to specific elements driving the induction of persistent arthralgia following MAYV infection. Furthermore, we have identified a subset of inflammatory cytokines that could serve as potential predictors for the development of persistent arthralgia. Future studies should evaluate the contributions of the immune response, and genetic polymorphisms among the human population and how they relate to the development of MAYV-induced persistent arthralgia. To date, few studies have attempted to genetically characterize MAYV isolates and obtain information about the evolution of MAYV in the Americas [51–53]. However, further studies are needed in order to fully characterize the genetic variations among MAYV strains recovered from patients that completely recovered from the disease and those developing persistent arthralgia. This information could yield critical knowledge to our understanding of MAYV pathogenesis.

Studies with CHIKV have reported variations in cytokine responses in patients with acute CHIKV infection residing in distinct geographic locations and thus it is possible that the immune cytokine and chemokine responses reported here might be specific to MAYV-infected patients from the Amazon basin of Peru. Nevertheless, a systemic meta-analysis of immune signatures in patients with acute CHIKV infection study was still able to find valuable cytokine markers that can distinguish CHIKV-infected individuals from those without CHIKV infection across patients from different geographic locations [41]. Thus, additional studies are needed to further validate the immune factors found to be induced during MAYV infection and to define the potential use of IL-13, IL-7 and VEGF as predictors of MAYV-disease severity. Future studies are also necessary to investigate whether MAYV infection induces persistent infection as has been suggested with CHIKV [23].

Current efforts to generate alphavirus vaccines have focused on the development of live-attenuated vaccines and their ability to elicit protective neutralizing antibody responses [54]. While this approach is of particular importance for pre-exposure protection, the possibility that live-attenuated vaccines may be capable of causing sustained pro-inflammatory responses or long-term arthralgia has not been addressed. Furthermore, the fact that neutralizing antibodies do not protect against severe disease outcomes suggest that studies are needed to assess potential live-attenuated vaccines for their ability to induce persistent inflammatory responses in innate immune cells. This will be critical for the development of safe alphavirus vaccines.

## Supporting Information

S1 Fig Kinetic profile of cytokine/chemokines in healthy donor control group from the Amazon basin of Peru. Serum samples were collected from healthy subjects and the serum concentrations of a variety of cytokines and chemokines were assessed by a multiplex cytokine bead array. Each symbol corresponds to the cytokine concentration of an individual subject and the solid horizontal line represents the mean cytokine concentration of the group.
(TIF)

S2 Fig Kinetic profile of cytokines and chemokines below the concentrations detected in healthy donor controls following MAYV infection. Serum samples were collected at the acute visit as well as the convalescent visit (20±10 days), at 90±10 days, at 180±15 days, and at 360±30 days after the acute visit. The serum concentrations of a variety of cytokines were assessed by a multiplex cytokine bead array. The box plot denotes the median, 25th percentile, and 75th percentile cytokine levels. The whiskers denote the minimum and maximum cytokine levels observed at each time point. The horizontal dotted line represents the median cytokine values for the healthy donor controls. Comparisons between MAYV infected subjects and healthy donors were performed by a 2-tailed Mann-Whitney test (*p<0.05).
(TIF)
