# Supplementary figures and images for: Long-Term Arthralgia after Mayaro Virus Infection Correlates with Sustained Pro-inflammatory Cytokine Response
Source: PLoS Negl Trop Dis. 2015 Oct 23;9(10):e0004104. doi: 10.1371/journal.pntd.0004104 (PMC4619727; doi:10.1371/journal.pntd.0004104)

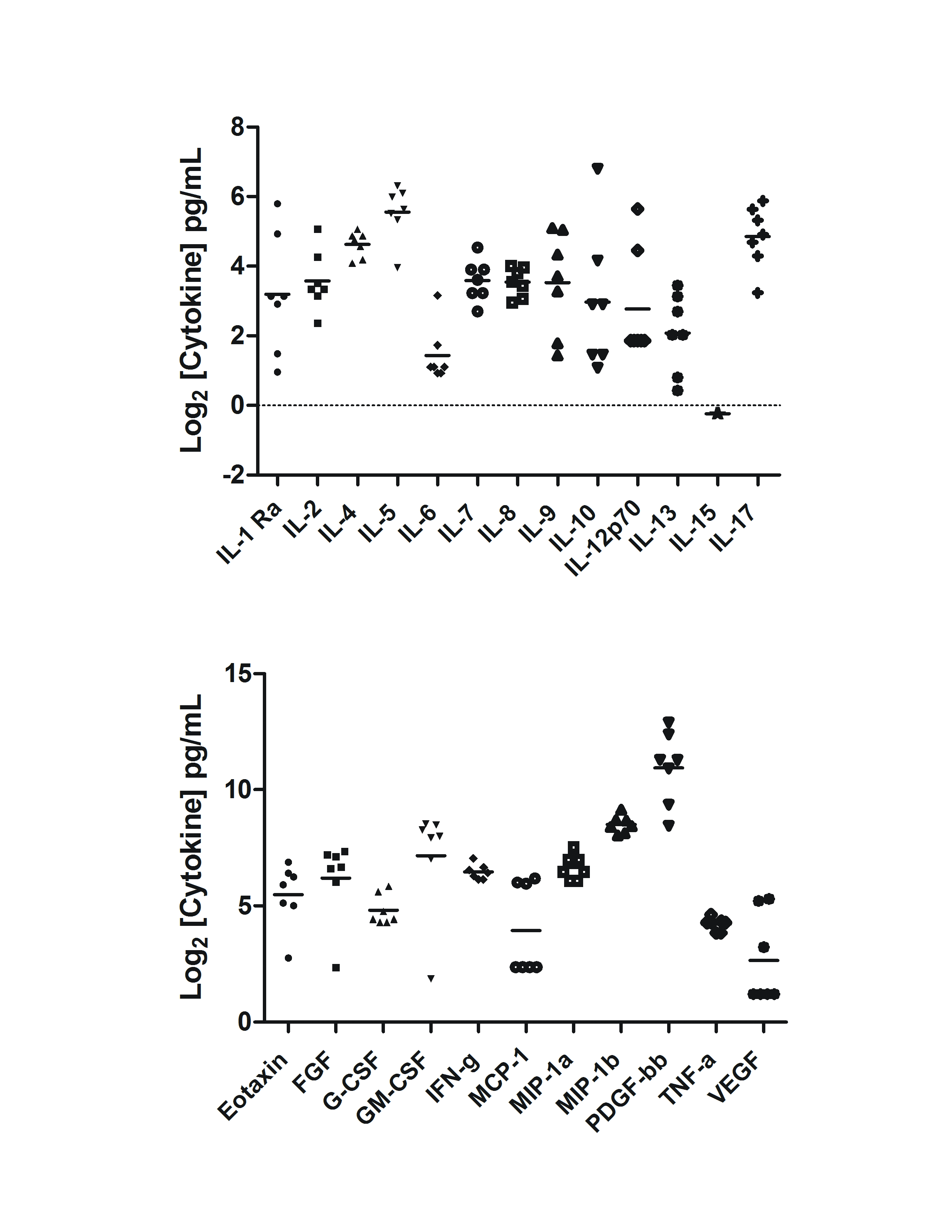

Supplement: S1 Fig — Serum samples were collected from healthy subjects and the serum concentrations of a variety of cytokines and chemokines were assessed by a multiplex cytokine bead array. Each symbol corresponds to the cytokine concentration of an individual subject and the solid horizontal line represents the mean cytokine concentration of the group. (TIF) [file pntd.0004104.s001.tif]

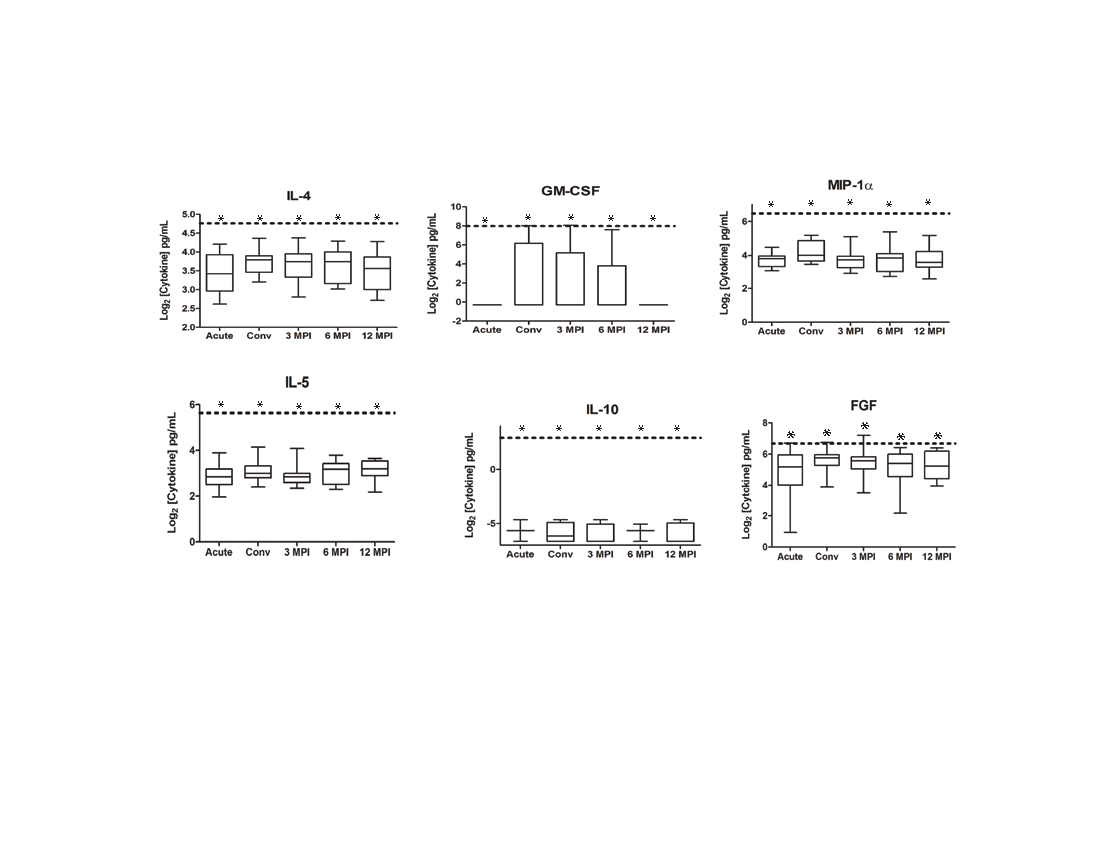

Supplement: S2 Fig — Serum samples were collected at the acute visit as well as the convalescent visit (20±10 days), at 90±10 days, at 180±15 days, and at 360±30 days after the acute visit. The serum concentrations of a variety of cytokines were assessed by a multiplex cytokine bead array. The box plot denotes the median, 25th percentile, and 75th percentile cytokine levels. The whiskers denote the minimum and maximum cytokine levels observed at each time point. The horizontal dotted line represents the median cytokine values for the healthy donor controls. Comparisons between MAYV infected subjects and healthy donors were performed by a 2-tailed Mann-Whitney test (*p<0.05). (TIF) [file pntd.0004104.s002.tif]
